# Comparative transcriptomic and metabolomic analysis reveals mechanisms of selenium-regulated anthocyanin synthesis in waxy maize (*Zea mays L.*)

**DOI:** 10.3389/fpls.2024.1466756

**Published:** 2024-10-03

**Authors:** Guangyu Guo, Yufeng Wang, Baoku Zhang, Haoran Yu, Liang Li, Guanglu Cao, Baicui Chen, Chengxin Li, Fanshan Bu, Song Teng, Qingtao Yu, Mingbo Gao, Baiwen Jiang, Kejun Yang

**Affiliations:** ^1^ College of Agriculture, Heilongjiang Bayi Agricultural University, Daqing, China; ^2^ Food and Cash Crops Branch, Harbin Academy of Agricultural Sciences, Harbin, China; ^3^ College of Resources and Environment, Northeast Agricultural University, Harbin, China

**Keywords:** *Zea mays L.*, selenium, anthocyanin biosynthesis, transcriptomic, metabolomic

## Abstract

Anthocyanins in maize (*Zea mays L.*) kernels determine the plant’s color and can enhance its resistance. Selenium (Se) significantly impacts plant growth, development, and secondary metabolic regulation. However, the molecular mechanisms by which Se regulates anthocyanin synthesis in waxy corn remain unclear. This study employed integrated transcriptomic and metabolomic analyses to investigate the mechanisms through which selenium influences anthocyanin synthesis in yellow and purple waxy corn. The results showed that maize varieties with higher anthocyanin content had higher selenium enrichment capacity in their kernels. Under selenium stress, HN2025 exhibited 1,904 more differentially expressed genes (DEGs) and 140 more differential metabolites compared to HN5. The expression levels of anthocyanin synthesis-related genes and transcription factors such as phenylalanine ammonia-lyase, flavonoid 3-hydroxylase (F3H), dihydroflavonol reductase (DFR), chalcone synthase (CHS), cinnamate-4-hydroxylase (C4H), anthocyanin 5,3-O-glucosyltransferases, and anthocyanidin reductase, MYB, and bHLH were strongly induced in HN2025. Metabolomic analysis revealed significant enrichment in anthocyanin biosynthesis, flavonoid and flavonol biosynthesis, glutathione metabolism, phenylalanine biosynthesis, and phenylalanine metabolism under selenium treatment. Three up-regulated PAL genes and one C4H gene were significantly enriched with DAMs in phenylalanine metabolism, phenylpropanoid biosynthesis, flavonoid biosynthesis, and anthocyanin biosynthesis, resulting in significant differences between HN5 and HN2025 in selenium-induced anthocyanin metabolism-related pathways. These findings provide a theoretical basis for understanding the effects of selenium on the molecular regulatory mechanisms of anthocyanin biosynthesis in maize kernels.

## Introduction

Maize (*Zea mays L.*) is a globally pivotal food crop, integral to agricultural production (Rosas-Castor et al., 2014). The anthocyanins present in maize grains are vital secondary metabolites, renowned for their potent antioxidant, anti-inflammatory, and anticancer properties (Xiao et al., 2023). These compounds not only determine the pigmentation of maize but also enhance the plant’s resistance to various environmental stresses (Yang et al., 2024). Selenium (Se), an essential trace element, plays a critical role in modulating plant growth, development, and secondary metabolism (Lu et al., 2020). Recent research indicates that selenium can regulate anthocyanin biosynthesis by modulating gene expression and metabolic pathways (Xu et al., 2015; Liu et al., 2021). Therefore, understanding the specific molecular mechanisms by which selenium influences anthocyanin synthesis in maize is of substantial importance.

Research has demonstrated that colored wheat grains exhibit superior Se absorption efficiency compared to common wheat (Xia et al., 2020). Due to their biological parallels, the interaction between Se and anthocyanins has been extensively studied. Post-Se treatment, the phenylalanine metabolism pathway is notably pronounced in green and purple wheat grains (Rothenberg et al., 2019; Wu et al., 2019). In these grains, Se significantly enhances anthocyanin synthesis, with R2R3-MYB and bHLH identified as crucial regulators of Se’s impact on anthocyanin biosynthesis (Pu et al., 2021). Selenium application has been shown to elevate the expression levels of UFGT and F3H genes involved in anthocyanin synthesis, thereby increasing anthocyanin accumulation in purple lettuce (Liu et al., 2017). These findings suggest that environmental factors influence anthocyanin accumulation by activating the expression of regulatory or structural genes within the anthocyanin synthesis pathway, which in turn modulate other cellular signaling pathways. Furthermore, the flavonoid synthesis pathway contains additional branches, such as proanthocyanidins, flavonols, and tannins, where synthesis rates decrease, thereby accelerating anthocyanin synthesis (Petrussa et al., 2013). In addition, selenium influences anthocyanin synthesis and accumulation through various mechanisms. For instance, Se treatment significantly upregulates the expression of key anthocyanin synthesis enzyme genes such as PAL, CHS, and DFR in tomato fruits, thereby enhancing anthocyanin biosynthesis (Wada et al., 2014; Zhang et al., 2022). In rice, Se promotes anthocyanin production by increasing the expression of critical genes in the anthocyanin pathway, including CHS, F3H, and ANS (Shih et al., 2008). Furthermore, Se treatment in strawberries boosts the activity of antioxidant enzymes like SOD, POD, and CAT, reduces reactive oxygen species (ROS) levels, alleviates oxidative stress, protects cell membrane integrity, and consequently promotes anthocyanin synthesis and accumulation (Liu et al., 2024). Genes associated with anthocyanin biosynthesis, phenylpropanoid biosynthesis, and flavonoid biosynthesis were significantly up-regulated after Se treatment, leading to the accumulation of anthocyanin metabolites in colored grain wheat (Xia et al., 2023).

Anthocyanin synthesis involves intricate metabolic pathways, and recent advancements have identified numerous genes and metabolites associated with this process through integrated transcriptomic and metabolomic approaches. In Chinese kale (*Brassica oleraceavar.alboglabra*), significant differences were observed in 23 critical anthocyanin biosynthesis genes, with association analyses indicating a strong correlation between *BoGSTF12* and anthocyanin levels (Tang et al., 2024). In Perilla, metabolomic and transcriptomic analyses under varying light intensities revealed that four key genes (CHI, 4CL, DFR, and ANS) and 147 transcription factors (including MYB, bHLH, bZIP, ERF, and NAC) play pivotal roles in the biosynthesis of malonyl isoflavones (Xie et al., 2022). Furthermore, in color-mutated sweetpotato (*Ipomoea batatas*), all 13 differentially accumulated metabolites were significantly down-regulated, with substantial reductions in anthocyanin metabolites and down-regulation of genes encoding key enzymes involved in the later stages of anthocyanin and flavonoid biosynthesis (Zhao et al., 2022). Moreover, both transcriptional and metabolic analyses have established that grape flesh color is significantly associated with C4H, CHS, and GST genes (Ju et al., 2023). In maize, several structural and regulatory genes implicated in anthocyanin synthesis have been reported, such as structural genes *ZmCHS* (Franken et al., 1991), *ZmCHI* (Grotewold and Peterson, 1994), *ZmF3H* (Deboo et al., 1995), and *ZmANS* (Weiss et al., 1993), as well as regulatory genes *P1* (Li et al., 2019), *C1* and *R1* (Riaz et al., 2019), and *RAC1* (Selinger and Chandler, 1999). The cloning of these genes has progressively elucidated the anthocyanin biosynthesis pathway. Despite extensive research on anthocyanins, their role in maize under selenium treatment remains largely unexplored. Further investigation is required to understand the molecular mechanisms through which selenium influences anthocyanin synthesis in waxy corn, offering potential insights into enhancing crop resilience and nutritional quality.

Anthocyanins and selenium, both recognized for their antioxidant properties, play essential roles in human health. Anthocyanins are prevalent in fruits and vegetables such as garlic, onions, and celery (Pyrzynska, 2009), which are also noted for their high selenium content. Prior research has indicated that pigmented crop varieties exhibit enhanced selenium concentrations and absorption rates, with exogenous selenium showing a linear relationship with anthocyanin content and diversity (Pu et al., 2021). Despite these findings, the regulatory relationship between selenium and anthocyanins in maize, especially at the transcriptional and metabolic levels, remains unclear. To address this deficiency, we exposed maize to three different exogenous selenium concentrations at various periods post-silking. We selected the concentration that demonstrated significant differences in selenium enrichment capacity and anthocyanin content across different maize varieties. Subsequently, we conducted transcriptomic and metabolomic analyses on maize kernels treated with this selenium concentration to elucidate the relationship between selenium and anthocyanins. This study aims to bridge the knowledge gap concerning the interactions between selenium and anthocyanins in waxy corn, thereby providing a theoretical foundation for future research in this area.

## Materials and methods

### Plant materials

In this study, we employed two waxy corn varieties as experimental materials: the yellow-waxy corn variety Hanian 5 (HN5) with a yellow seed coat and the purple-waxy corn variety Hanuo 2025 (HN2025) with a purple seed coat, with the embryo and endosperm of HN5 being yellow and creamy-white, and that of HN2025 creamy-white. HN5 is derived from the crossbreeding of the maternal HA4146 and paternal HA4139, and HN2025 is derived from HA1303 and HA3165. Both varieties were developed by the Agricultural Science Research Institute in Harbin, Heilongjiang Province, China. The experiments were conducted at the Harbin Academy of Agricultural Sciences (126.47′E, 45.85′N). The soil type at the site was black soil with the following nutrient contents: total nitrogen 0.92 g·kg^-1^, available nitrogen 45.3 mg·kg^-1^, available phosphorus 17.9 mg·kg^-1^, available potassium 93.7 mg·kg^-1^, and a pH of 6.85. Baseline soil selenium contents for the years 2022, 2023, and 2024 were 0.151 mg/kg, 0.136 mg/kg, and 0.145 mg/kg, respectively. Referring to the local standard ‘Requirements for Evaluation of Selenium-rich Soil’ in Heilongjiang Province, China, the test field was classified as low selenium - selenium-deficient soil.

### Field experiment design

The field experiment was structured using a two-factor split-plot design with a planting density of 54,000 plants per hectare. The primary plots were subjected to four levels of exogenous selenium application (selenium-enriched fertilizer): 0 (control), 11.25, 22.5, and 33.75 g·hm^-2^, labeled as Se0, Se1, Se2, and Se3, respectively. The Se0 plots were sprayed with an equivalent volume of deionized water to serve as controls. The selenium-enriched fertilizer was supplied by Harbin Green Oasis Star Biotechnology Co., Ltd. (Registration number: Microbial Fertilizer (2021) No. 10072, Standard: GB20287-2006, Formulation: Aqueous solution). The subplots received selenium treatments at three specific growth stages: the silking stage (S0), five days post-silking (S5), and ten days post-silking (S10). All treatments were administered on clear days with no expected rainfall within 24 hours, between 16:00 and 18:00. Selenium fertilizer treatments were carried out using DELIXI manual motorized sprays with the same nozzles throughout. Each treatment was replicated biologically three times to ensure the reliability and reproducibility of the results.

### Phenotype measurement

To determine the total selenium content, a 3.0 g sample of dried material was weighed and placed in a digestion vessel. Subsequently, 10 mL of a nitric acid-perchloric acid mixture was added to the sample. The vessel was then covered with a watch glass and allowed to digest overnight at room temperature. The following day, the mixture was heated on a hot plate, with additional nitric acid added as necessary. Heating continued until the solution turned clear and colorless, emitting white fumes, and the remaining volume was reduced to approximately 2 mL. After cooling, 5 mL of hydrochloric acid was added, and the mixture was reheated until it again became clear and colorless with white fumes. The solution was then removed from the heat, allowed to cool to room temperature, and transferred to a 10 mL volumetric flask. Approximately 2.5 mL of potassium ferricyanide solution was added, and the solution was diluted to 50 mL with distilled water, mixed thoroughly, and filtered prior to analysis. The total selenium concentration in the prepared samples was determined using Hydride Generation-Atomic Fluorescence Spectrometry (HG-AFS 9120, Beijing Puxi). This procedure was conducted by the national standard method GB5009.93-2017, titled “Determination of Selenium in Food”.

Inorganic selenium content was determined using Hydride Generation-Atomic Fluorescence Spectrometry (HG-AFS). To prepare the samples, 20 mL of 50% hydrochloric acid was added to each sample tube, followed by thorough mixing and ultrasonic treatment for 45 minutes. The mixture was then transferred to a boiling water bath for 30 minutes. After cooling, the mixture was heated until the volume was reduced to 2 mL. Subsequently, the solution was cooled again, and 5 mL of 50% hydrochloric acid was added. The mixture was evaporated to 2 mL and cooled once more. Finally, 5 mL of concentrated hydrochloric acid was added, and the solution was diluted to a final volume of 25 mL with purified water. The prepared solution was then used to measure the inorganic selenium content. The organic selenium content was calculated using the formula: Organic Selenium = Total Selenium Content - Inorganic Selenium Content.

Anthocyanin content was determined using the pH differential method. Initially, 0.2 g of sample was weighed and 2 mL of acetone was added. The mixture was shaken well and centrifuged, with the supernatant discarded. Next, 2 mL of 0.1% HCl was added, and the mixture was placed in a 60°C water bath for 1 hour. After heating, the sample was centrifuged and the supernatant was retained. The heating and centrifugation steps were repeated, and the supernatants were combined. A 1 mL aliquot of the combined supernatant was transferred to a 10 mL centrifuge tube, and 5 mL each of pH 1.0 HCl-KCl buffer and pH 4.5 HAc-NaAc buffer were added. The mixture was thoroughly mixed, and distilled water was used as a control. Absorbance was measured at 510 nm and 700 nm wavelengths using a spectrophotometer to determine anthocyanin content.

### RNA extraction and transcriptome sequencing analysis under selenium treatment

Maize was grown to the fresh consumption stage after 18 days of 22.5 g/hm² selenium fertilizer treatment, samples were collected using a five-point sampling method. Within each plot, 10 representative ears of glutinous corn, characterized by uniform growth and similar ear morphology, were selected. After shelling, the kernels were thoroughly mixed and prepared for subsequent analysis. Fresh kernels from HN5 and HN2025, treated with 22.5 g/hm² selenium fertilizer five days post-silking, were flash-frozen in liquid nitrogen. Each treatment was replicated biologically three times. These kernels were then sealed in 10 mL centrifuge tubes and stored at -80°C for subsequent transcriptome and metabolome analyses. Total RNA was isolated using TRIzol reagent (Invitrogen, Carlsbad, CA, USA). Following extraction, mRNA was purified with the NucleoSpin RNA Clean-up kit (MACHEREY-NAGEL, Düren, Germany) according to the manufacturer’s protocol. The purified mRNA was then fragmented and reverse-transcribed into cDNA using random hexamer primers. The second strand of cDNA was synthesized using DNA polymerase I, RNase H, dNTPs, and a specific buffer. The resulting cDNA fragments were purified using a QiaQuick PCR extraction kit, end-repaired, polyadenylated, and ligated to Illumina sequencing adapters. These ligation products were then size-selected through 1% agarose gel electrophoresis, PCR-amplified, and sequenced using an Illumina HiSeqTM 2500 (Wuhan, China).

Transcripts were assembled using Cufflinks to identify both known and novel transcripts. Stringent quality control measures were implemented to ensure the accuracy and integrity of the data prior to analysis. The filtering criteria included: (1) removal of reads containing adapters, (2) removal of paired reads if the N content in any sequencing read exceeded 10% of the total bases, and (3) removal of paired reads if more than 50% of the bases were of low quality (Q ≤ 20). Differential expression analysis was performed across samples to identify differentially expressed genes (DEGs) with a false discovery rate (FDR) < 0.05 and |log2FC| > 1. Identified genes were annotated against the Gene Ontology (GO) (available online: http://www.r-project.org/) and Kyoto Encyclopedia of Genes and Genomes (KEGG) (available online: http://kobas.cbi.pku.edu.cn/) databases to determine their functions. Significant functional categories and metabolic pathways among DEGs were identified with an FDR ≤ 0.05, providing insights into the biological processes and pathways influenced by selenium treatment.

### Liquid chromatography electrospray ionization tandem mass spectrometry

The sample extracts were subjected to analysis using an LC-ESI-MS/MS system, comprising an HPLC (Shim-pack UFLC SHIMADZU CBM30A system) and a mass spectrometer (Applied Biosystems 4500 Q TRAP). For liquid chromatography, 5 µL of each sample was injected into a Waters ACQUITY UPLC HSS T3 C18 column (1.8 µm, 2.1 mm x 100 mm). The mobile phase consisted of 0.04% acetic acid in acetonitrile (solvent B) and 0.04% acetic acid in Milli-Q water (solvent A). The gradient elution program was as follows: the initial condition of 5% solvent B was maintained, followed by a linear increase to 95% solvent B over 11 minutes. This was held at 95% solvent B for 1 minute, then rapidly decreased to 5% solvent B within 0.1 minute, and maintained at 5% solvent B for an additional 3 minutes. The flow rate was set at 0.40 mL/min, and the column temperature was kept constant at 40°C to ensure optimal separation and reproducibility.

Mass spectrometry analysis was conducted using an API 4500 Q TRAP LC/MS/MS system. Instrument tuning and mass calibration were executed using polypropylene glycol solutions at concentrations of 10 μmol/L and 100 μmol/L. QQQ scans were performed via multiple reaction monitoring (MRM) experiments, utilizing nitrogen as the collision gas at a pressure of 5 psi. The declustering potential (DP) and collision energy (CE) were optimized specifically for each MRM transition, which was monitored according to the elution time of the metabolites (Zou et al., 2020). This rigorous method ensured precise quantification and identification of metabolites, adhering to the highest standards of scientific accuracy and reproducibility (Zou et al., 2020).

### Quantitative reverse-transcription PCR

Quantitative reverse transcription PCR (qRT-PCR) was performed as previously described (Livak and Schmittgen, 2001). All samples used for qRT-PCR were derived from transcriptome sequenced samples. Total RNA was isolated from the samples using the MAGEN RNA extraction kit (MAGEN, USA) and reverse-transcribed using the Evo M-MLV RT kit (Accurate, Hunan, China). Primers were designed using Primer 5.0, with sequences listed in **Supplementary Table S1**. PCR amplification was conducted using the LightCycler 480 II Real-Time PCR system (Roche, Basel, Switzerland), with the maize Actin gene serving as the internal control.

### Statistical analysis

One-way analysis of variance (ANOVA) was conducted using SPSS 22.0 software (Softonic International, Barcelona, Spain) to evaluate the differences among various treatments.

## Results

### Phenotypic detection of selenium and anthocyanin content in maize kernels

**Figure 1:**
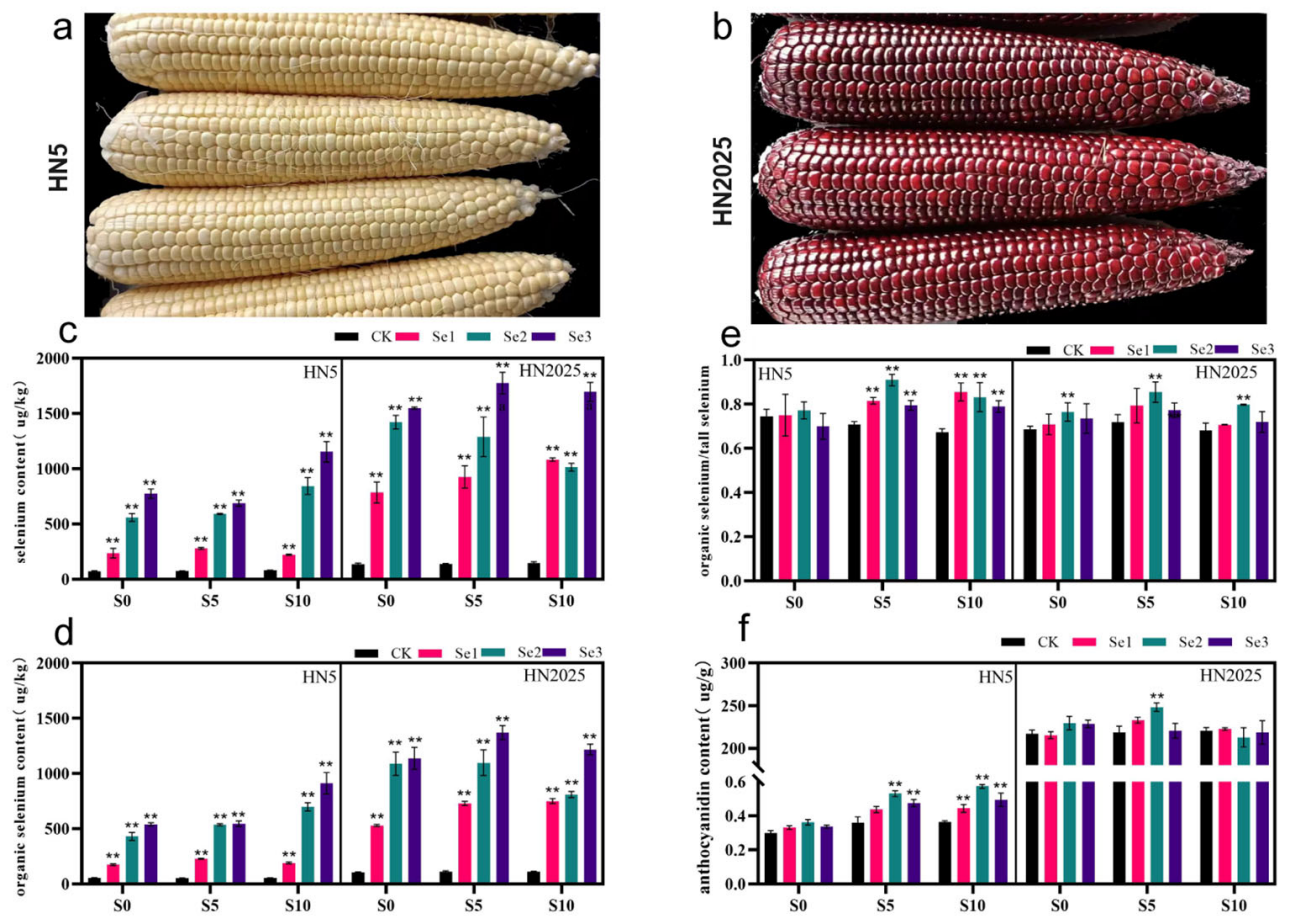
Phenotype, selenium and anthocyanin content of HN5 and HN2025. **(A)** HN5 (Hanian5); **(B)** HN2025(Hanuo2025); **(C)** Distribution of total selenium content in HN5 and HN2025 at the S0, S5 and S10 stage in the 11.25, 22.5, and 33.75 g/hm^2^ selenium concentration treatments; **(D)** Distribution of organic selenium content in HN5 and HN2025 at the S0, S5 and S10 stage in the 11.25, 22.5, and 33.75 g/hm^2^ selenium concentration treatments; **(E)** Organic selenium as a proportion of total selenium; **(F)** Distribution of anthocyanin content in HN5 and HN2025 at the S0, S5 and S10 stage in the 11.25, 22.5, and 33.75 g/hm^2^ selenium concentration treatments. ** represents the level of difference between the treatment and control groups at P < 0.01.

HN5 and HN2025 are waxy corn with yellow and purple seed coat, respectively (**Figures 1A, B**). We examined the selenium and anthocyanin contents of these varieties following treatments with various selenium concentrations at the S0, S5, and S10 periods. Our findings revealed that, compared to the control, the total and organic selenium contents of HN5 and HN2025 seeds significantly differed under the 11.25, 22.5, and 33.75 g/hm² selenium treatments (**Figures 1C, D**). Selenium accumulation in the plants increased with higher selenium concentrations. However, at the S10 stage, the total and organic selenium contents of HN2025 showed a decreasing trend. Notably, the highest relative organic selenium content was observed at the S5 growth stage under the 22.5 g/hm² selenium concentration, indicating that the plant’s selenium enrichment capacity peaked at this stage (**Figure 1E**). Furthermore, under this specific treatment condition, the anthocyanin content of HN2025 exhibited significant differences compared to other treatments. In contrast, HN5, which inherently has lower anthocyanin content (**Figure 1F**), accumulated more anthocyanins with the extension of the growth stage compared to the control. This indicates a differential response in selenium and anthocyanin accumulation between the two varieties, highlighting the complex interplay between selenium treatment and anthocyanin biosynthesis.

### Differential analysis of HN5 and HN2025 at the transcriptome level under selenium treatment

**Figure 2:**
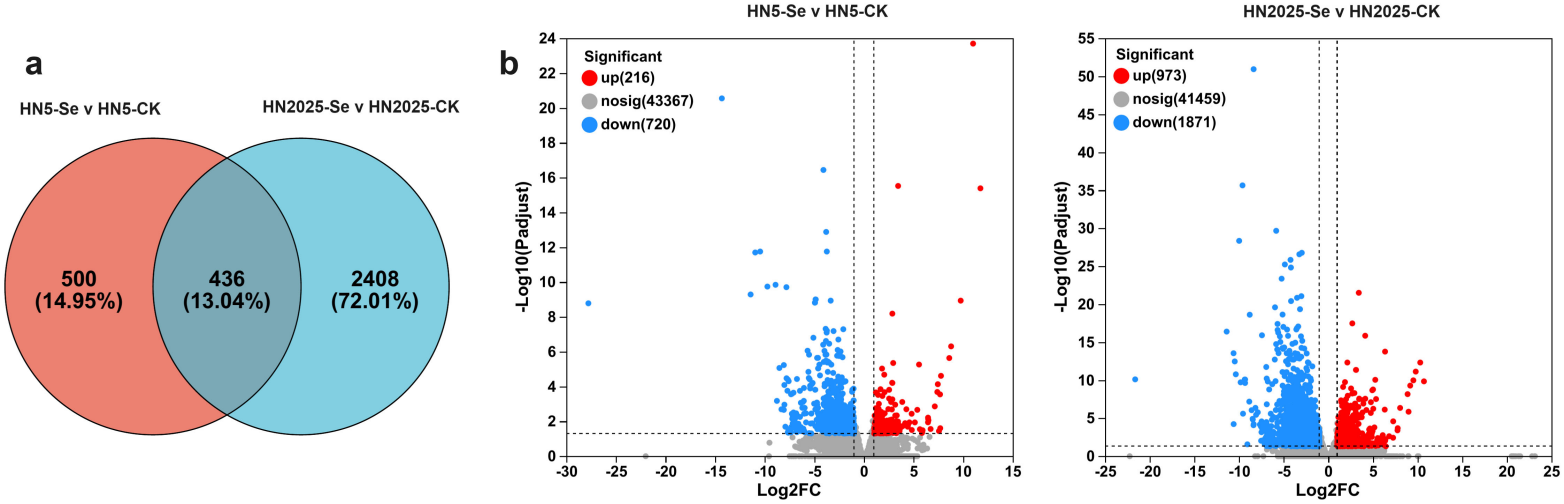
Differentially expressed genes in HN5 and HN2025 under selenium conditions. **(A)** Venn diagram showing the number of DEGs detected in HN5 and HN2025 under selenium conditions; **(B)** Heat maps showing differentially expressed genes in HN5-Se vs. HN5-CK, and HN2025-Se vs. HN2025-CK. CK for the control group, Se for the selenium-treated, and Nosig for no significance.

Under selenium treatment, significant physiological differences were observed between HN5 and HN2025. To elucidate the regulatory mechanisms of selenium treatment and identify novel regulatory factors, RNA-seq was employed to investigate the transcriptomic changes in the leaves of HN5 and HN2025 subjected to selenium treatment. Differentially expressed genes (DEGs) were quantitatively analyzed using the DESeq2 package in R. A total of 3,344 DEGs were detected under selenium treatment, with 436 DEGs showing differential expression in both varieties. Additionally, 2,408 and 500 genes were uniquely expressed in HN2025 and HN5, respectively (**Supplementary Tables S2, S3**). Among the 436 common DEGs, 52 genes in HN2025 exhibited significantly higher differential expression levels compared to HN5. These 52 genes, along with the 2,408 uniquely DEGs in HN2025, are likely to play a pivotal role in maize’s response to selenium treatment (**Figure 2**). This includes several genes directly involved in the anthocyanin synthesis pathway, such as phenylalanine ammonia-lyase (PAL) *Zm00001eb247620*, *Zm00001eb077230*, and *Zm00001eb185240*; flavanone 3-hydroxylase (F3H) *Zm00001eb017350*; dihydroflavonol reductase (DFR) *Zm00001eb344520* and *Zm00001eb317040*; chalcone synthase (CHS) *Zm00001eb198050*; cinnamate 4-hydroxylase (C4H) *Zm00001eb240910*; anthocyanidin 5,3-O-glucosyltransferase-like *Zm00001eb154000*; and anthocyanidin reductase1 *Zm00001eb431500* (**Supplementary Table S2**). The expression of these DEGs due to selenium treatment exclusively in HN2025 suggests that selenium treatment induces the expression of genes related to anthocyanin metabolic pathways more significantly in varieties highly enriched in anthocyanins.

### GO and KEGG enrichment analysis

**Figure 3:**
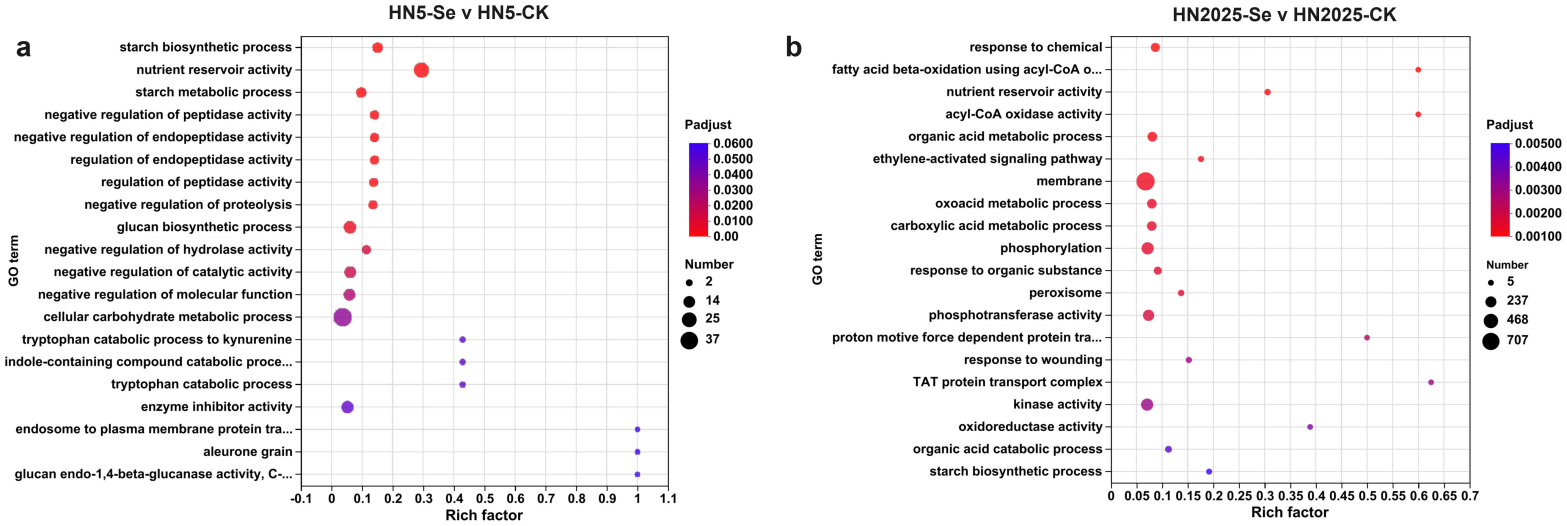
The Gene Ontology (GO) enrichment analysis under selenium conditions. **(A)** the top 20 significantly rich GO terms in HN5-Se vs. HN5-CK; **(B)** the top 20 significantly rich GO terms in HN2025-Se vs. HN2025-CK. CK for the control group, and Se for the selenium-treated. Rich factor: Ratio of the number of genes/transcripts enriched in the pathway (Sample number) to the number of annotated genes/transcripts (Background number). The larger the value of rich factor, the greater the degree of enrichment. Padjust: p-value corrected using the BH method.

To elucidate the functional roles of the DEGs and their involvement in relevant biological processes, we conducted Gene Ontology (GO) and Kyoto Encyclopedia of Genes and Genomes (KEGG) enrichment analyses. The GO enrichment analysis revealed a differential enrichment of GO terms across various comparisons. Specifically, the number of enriched DEGs in GO terms for the comparisons HN5-Se vs. HN5-CK and HN2025-Se vs. HN2025-CK were 812 and 2,415, respectively (**Supplementary Tables S4, S5**). Annotation analysis identified 18 GO terms closely associated with anthocyanin biosynthesis, primarily within the Biological Process (BP) and Molecular Function (MF) categories. In the HN5-Se vs. HN5-CK comparison, the relevant GO terms in the MF category included GO:0016661 and GO:0016664 (oxidoreductase activity). In the HN2025-Se vs. HN2025-CK comparison, the relevant GO terms in the BP category included GO:0006559 (L-phenylalanine catabolic process), GO:0009800 (cinnamic acid biosynthetic process), GO:0009803 (cinnamic acid metabolic process), and GO:2000762 (regulation of phenylpropanoid metabolic process). In the MF category, 12 GO terms (GO:0016491, GO:0016624, GO:0016634, GO:0016645, GO:0016647, GO:0016701, GO:0016702, GO:0016705, GO:0016716, GO:0016722, GO:0052894, GO:0052901) were identified as related to oxidative activity. Additionally, the top 20 most significantly enriched GO terms coincided in only two entries in both groups (**Figure 3**).

**Figure 4:**
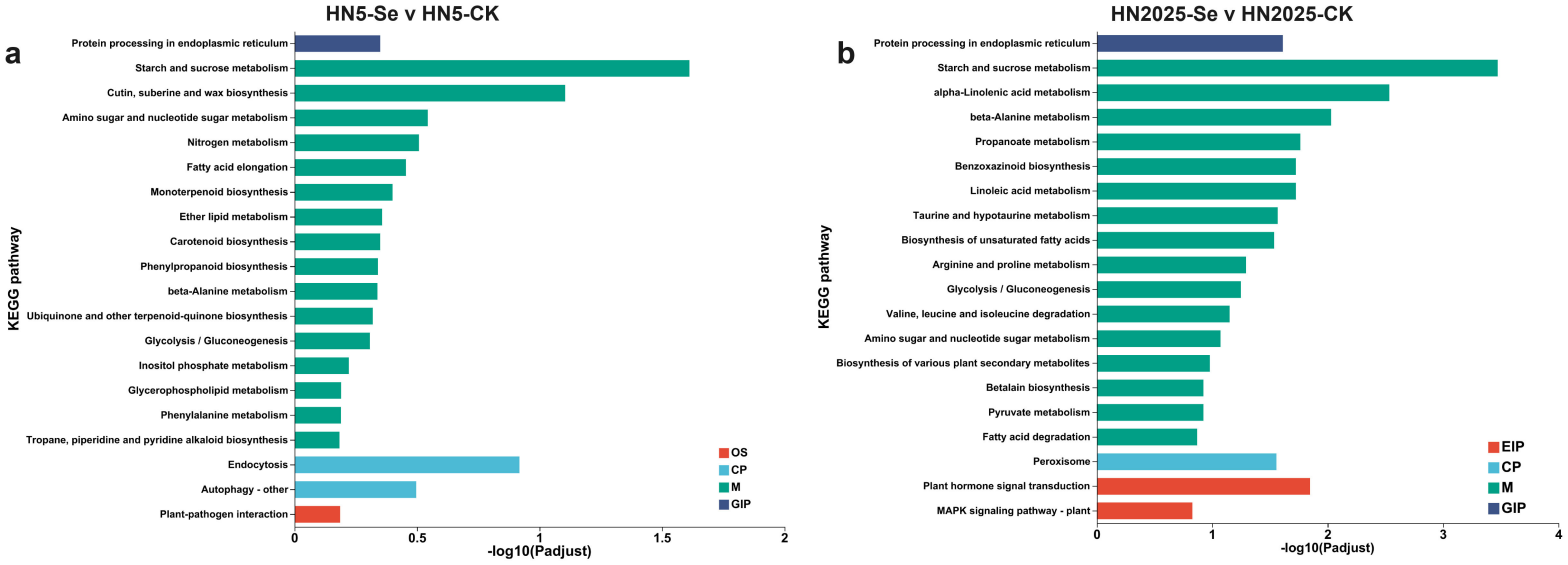
The Kyoto Encyclopedia of Genes and Genomes (KEGG) enrichment analysis under selenium conditions. **(A)** the top 20 signifcantly rich KEGG terms in HN5-Se vs. HN5-CK; **(B)** the top 20 signifcantly rich KEGG terms in HN2025-Se vs. HN2025-CK. CK for the control group, and Se for the selenium-treated. Different colors in the same comparison group indicate different branches of the KEGG metabolic pathway, namely metabolism (M), genetic information processing (GIP), environmental information processing (EIP), cellular processes (CP), and organismal systems (OS), respectively.

The KEGG enrichment analyses for the comparisons HN5-Se vs. HN5-CK and HN2025-Se vs. HN2025-CK revealed the involvement of 98 and 122 significantly different metabolic pathways, respectively (**Supplementary Tables S6, S7**). Among these, six pathways in HN5-Se vs. HN5-CK were significantly associated with anthocyanin biosynthesis: Flavonoid biosynthesis (map00941), Phenylalanine biosynthesis (map00400), Flavone and flavonol biosynthesis (map00944), Phenylpropanoid biosynthesis (map00940), Phenylalanine metabolism (map00360), and Circadian rhythm - plant (map04712). In addition to these six metabolic pathways, Isoflavonoid biosynthesis (map00940) was identified in HN2025-Se vs. HN2025-CK. Phenylpropanoid biosynthesis exhibited the highest gene enrichment in all comparisons, with 10 and 17 genes, respectively. The highest enrichment factor for Isoflavonoid biosynthesis was 0.33. The structural genes uniquely identified in the HN2025-Se vs. HN2025-CK comparison group included PAL (*Zm00001eb247620*, *Zm00001eb185240*, *Zm00001eb077230*), CHS (*Zm00001eb198050*), C4H (*Zm00001eb240910*), and anthocyanidin reductase1 (*Zm00001eb431500*). Furthermore, the top 20 significantly enriched pathways were primarily associated with carbohydrate metabolism, amino acid metabolism, lipid metabolism, MAPK signaling pathway, plant-pathogen interaction, and hormone signal transduction (**Figure 4**).

### Differential expression analysis of transcription factors related to anthocyanin biosynthesis under selenium treatment

The synthesis of anthocyanins is intricately regulated by the expression of various regulatory genes, prominently featuring transcription factors from the MYB family, bHLH family, WD40 repeat proteins, and PAP1-LIKE (PPL) proteins. These transcription factors typically form complexes to co-regulate the expression of genes involved in the anthocyanin biosynthesis pathway (Xu et al., 2015; Liu et al., 2021). Our study found that selenium treatment led to the differential expression of 8 MYB and 3 bHLH genes in the HN5-Se vs. HN5-CK comparison, and 19 MYB and 16 bHLH genes in the HN2025-Se vs. HN2025-CK comparison (**Supplementary Table S8**). These DEGs were enriched in several key signaling pathways, including the PI3K-Akt signaling pathway (map04151), the MAPK signaling pathway in plants (map04016), and plant hormone signal transduction (map04075). Notably, the genes *Zm00001eb124630*, *Zm00001eb142500*, *Zm00001eb179590*, and *Zm00001eb366540* were down-regulated in both varieties. The remaining 31 genes were uniquely expressed in HN2025, indicating a higher responsiveness of MYB and bHLH transcription factors to selenium treatment in the anthocyanin-rich variety HN2025. These 31 transcription factors are likely to play crucial upstream regulatory roles in the anthocyanin synthesis pathway, enhancing our understanding of the molecular mechanisms underpinning selenium-induced anthocyanin biosynthesis.

### Metabolomic analysis of HN5 and HN2025 under selenium treatment

**Figure 5:**
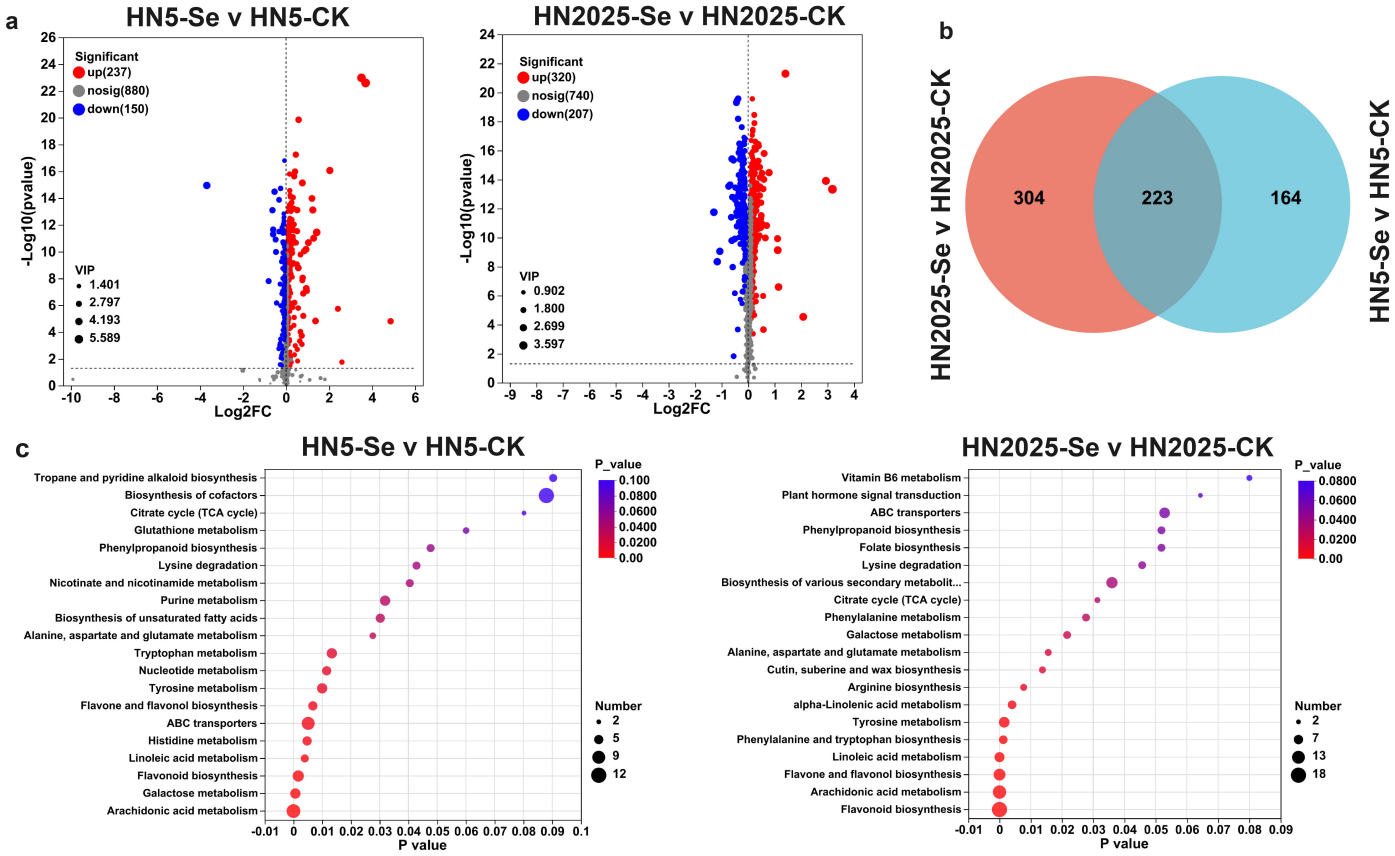
Differential metabolite analysis. **(A)** Manhattan diagram of metabolites of HN5-Se vs. HN5-CK and HN2025-Se vs. HN2025-CK; **(B)** Venn diagram showing the number of DAMs detected in HN5 and HN2025 under selenium conditions. **(C)** The top 20 signifcantly rich KEGG terms of DAMs in HN5-Se vs. HN5-CK and HN2025-Se vs. HN2025-CK. CK for the control group, and Se for the selenium-treated. Rich factor: Ratio of the number of genes/transcripts enriched in the pathway (Sample number) to the number of annotated genes/transcripts (Background number). The larger the value of rich factor, the greater the degree of enrichment.

The transcriptome analysis of two maize varieties indicates that selenium treatment may activate various metabolic pathways involved in anthocyanin regulation. To corroborate these findings at the metabolomic level, we conducted a comprehensive analysis of the metabolite profiles of both varieties with and without selenium treatment. Selenium treatment resulted in the identification of 387 differentially accumulated metabolites (DAMs) in the HN5-Se vs. HN5-CK comparison, with 237 metabolites upregulated and 150 downregulated. In the HN2025-Se vs. HN2025-CK comparison, 527 DAMs were identified, comprising 320 upregulated and 207 downregulated metabolites (**Figure 5A**; **Supplementary Tables S9, S10**). Among these DAMs, 223 were common to both comparisons, with 141 upregulated and 81 downregulated in both groups (**Figure 5B**). Additionally, in the HN2025-Se vs. HN2025-CK comparison, 304 DAMs were uniquely expressed. These include critical compounds in the anthocyanin biosynthesis pathway, such as naringenin, narirutin, kaempferitrin, cinnamic acid, L-phenylalanine, quercetin-3-O-glucosyl-6’’-acetate, apigenin, and intermediates like coumaroyl and chalcone.

KEGG enrichment analysis revealed that selenium treatment significantly enriched 64 metabolic pathways in both maize varieties, among which pathways related to anthocyanin biosynthesis included anthocyanin biosynthesis, flavone and flavonol biosynthesis, glutathione metabolism, phenylalanine biosynthesis, and phenylalanine metabolism (**Supplementary Tables S11, S12**). Notably, these 64 metabolic pathways encompassed 232 DAMs in the HN2025-Se vs. HN2025-CK comparison, compared to 184 in the HN5-Se vs. HN5-CK comparison, indicating a more pronounced impact of selenium treatment on the physiological metabolism of HN2025. Among the top 20 most significantly enriched pathways, eight were common to both groups, including “Galactose metabolism,” “Alanine, aspartate and glutamate metabolism,” “Flavone and flavonol biosynthesis,” “Lysine degradation,” “Flavonoid biosynthesis,” and “Arachidonic acid metabolism” (**Figure 5C**). Unique to HN2025 were pathways such as Butanoate metabolism, Ether lipid metabolism, Fructose and mannose metabolism, Glycolysis/Gluconeogenesis, and Pyruvate metabolism. These biological pathways typically influence anthocyanin synthesis by affecting phenylpropanoid metabolism through sugar metabolism or by providing energy for anthocyanin precursor synthesis. The unique enrichment of these pathways in HN2025 may elucidate the differences in anthocyanin synthesis between the two maize varieties, highlighting the specific metabolic adjustments contributing to the enhanced anthocyanin accumulation in response to selenium treatment.

### Integrated transcriptomic and metabolomic analysis

**Figure 6:**
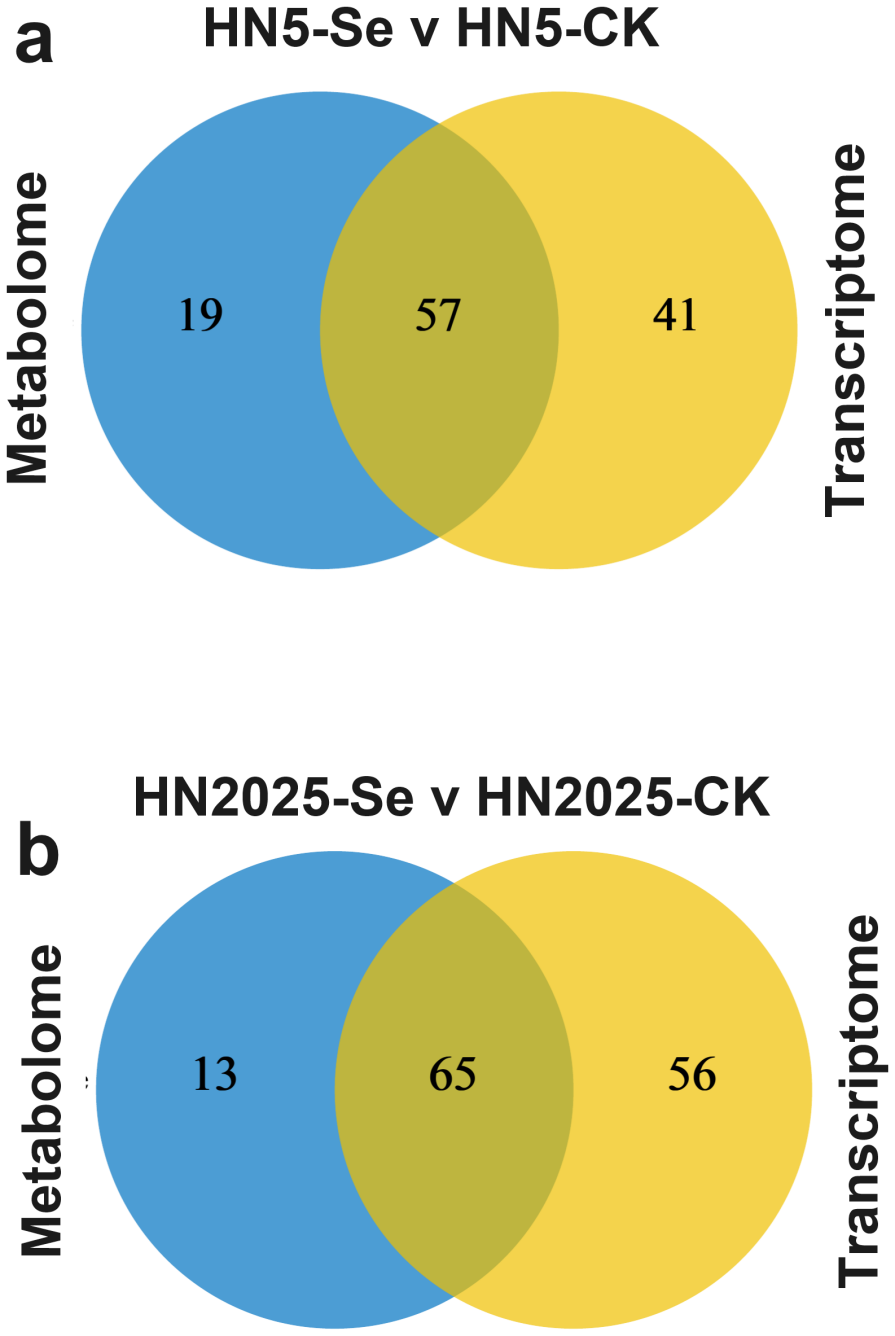
Joint analysis of the transcriptome and metabolome. **(A)** Venn diagram showing the number of metabolic pathways enriched to DEG and DAM in HN5-Se vs. HN5-CK. **(B)** Venn diagram showing the number of metabolic pathways enriched to DEG and DAM in HN2025-Se vs. HN2025-CK. CK for the control group, and Se for the selenium-treated.

**Figure 7:**
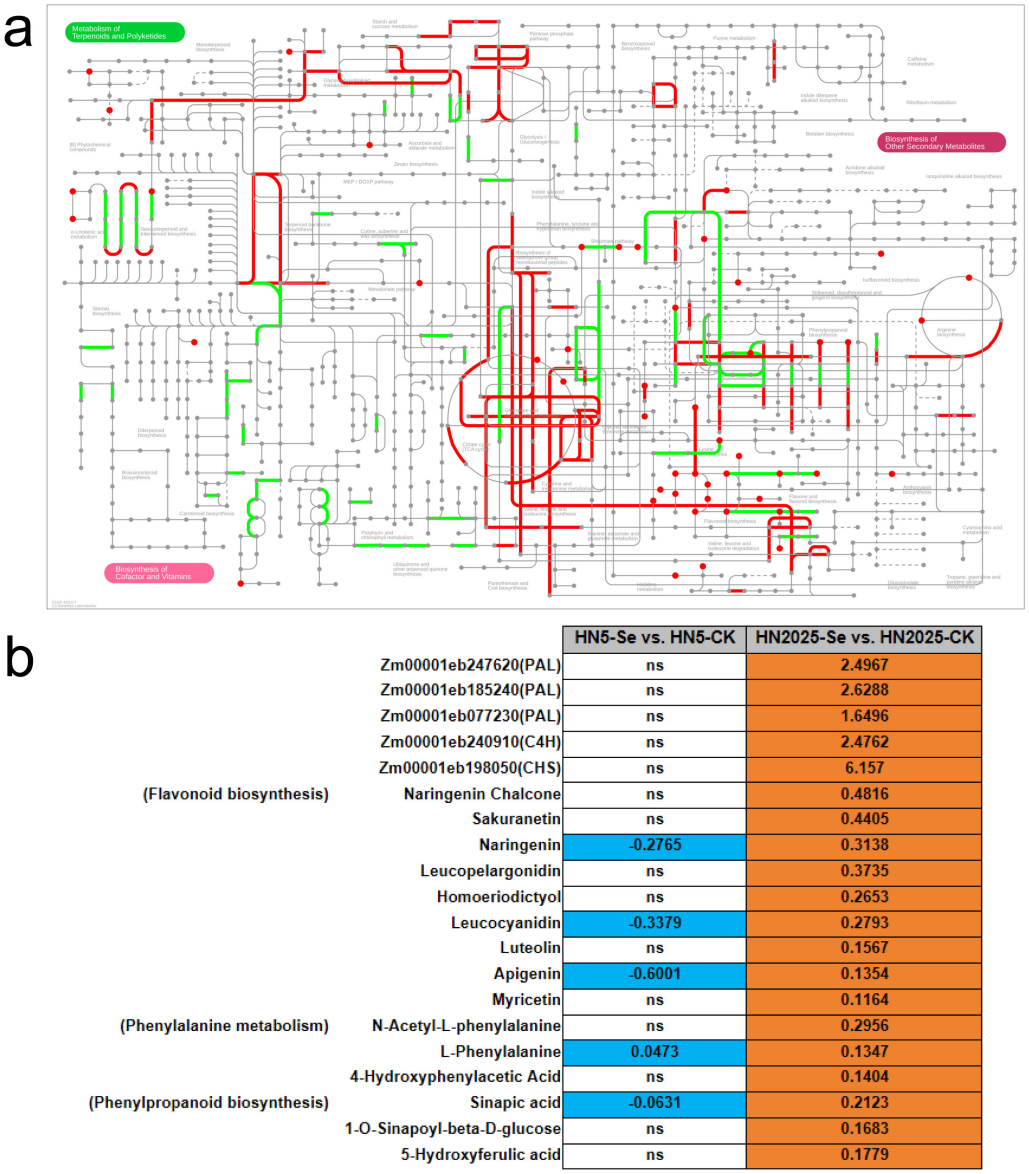
Pathway analysis between DEG and DAM in maize under selenium treatment. **(A)** Pathway annotation information for metabolites and genes, red represents metabolite-annotated pathways, green represents gene-annotated pathways. **(B)** DEG and DAM in the anthocyanin synthesis pathway are summarised, with ns representing non-significant.

To further elucidate the regulatory mechanisms of anthocyanin biosynthesis under selenium treatment, we conducted an integrated transcriptomic and metabolomic analysis to explore the correlation between anthocyanin metabolites and gene expression profiles. KEGG annotation of DEGs and DAMs revealed that in HN5-Se vs. HN5-CK, post-selenium treatment, DEGs and DAMs were jointly mapped to 57 metabolic pathways (**Supplementary Table S13**; **Figure 6A**). These pathways included Phenylalanine metabolism, Phenylalanine, tyrosine, and tryptophan biosynthesis, Glutathione metabolism, and Flavonoid biosynthesis, all of which are directly implicated in anthocyanin synthesis. In the HN2025-Se vs. HN2025-CK comparison, selenium treatment resulted in DEGs and DAMs being annotated to 65 metabolic pathways (**Supplementary Table S14**; **Figure 6B**), including the aforementioned four pathways related to anthocyanin synthesis. Notably, there was a higher number of DEGs and DAMs in these pathways for HN2025-Se vs. HN2025-CK, indicating that the high-anthocyanin variety HN2025 may involve more complex physiological and metabolic responses to selenium treatment compared to HN5. We constructed a pathway map summarizing annotated metabolites and genes (**Figure 7**; **Supplementary Table S15**), highlighting the significant impact of selenium stress on secondary metabolism, including phenylalanine metabolism, flavonoid metabolism, anthocyanin metabolism, and the biosynthesis of flavones and flavonols (**Figure 7A**). These metabolic pathways exhibited an overall upregulation of metabolites in HN2025, whereas in HN5, they were generally downregulated or showed no significant change following selenium treatment. This suggests that selenium treatment induces distinct metabolic networks upstream of anthocyanin biosynthesis in HN2025 and HN5, emphasizing the differential regulatory mechanisms in response to selenium stress (**Figure 7B**).

**Figure 8:**
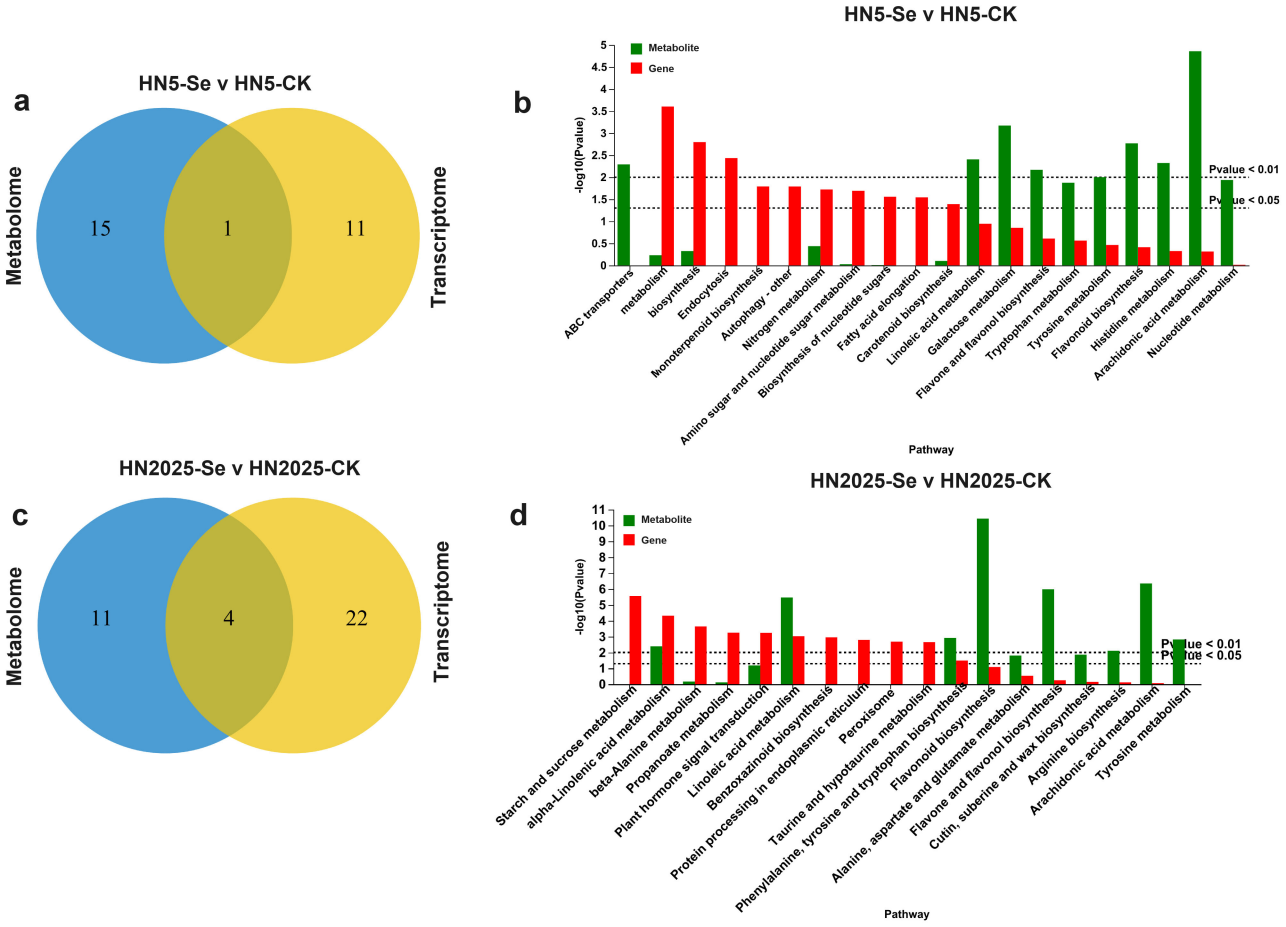
Comparative analysis of significantly enriched DEG and DAM. **(A)** Venn diagrams for pathways significantly enriched in gene set (default P<0.05) and pathways significantly enriched in metabolic set (default P<0.05) in HN5-Se vs. HN5-CK; **(B)** The bar charts show the pathways significantly enriched in gene set and the pathways significantly enriched in metabolite set in HN5-Se vs. HN5-CK, the red bars are the result of gene enrichment and the green bars are the result of metabolite annotation; **(C)** Venn diagrams for pathways significantly enriched in gene set (default P<0.05) and pathways significantly enriched in metabolic set (default P<0.05) in HN2025-Se vs. HN2025-CK; **(D)** The bar charts show the pathways significantly enriched in gene set and the pathways significantly enriched in metabolite set in HN2025-Se vs. HN2025-CK. CK for the control group, and Se for the selenium-treated.

Further KEGG enrichment analysis revealed significant pathway enrichment for DEGs and DAMs in HN5-Se vs. HN5-CK and HN2025-Se vs. HN2025-CK. In the HN5-Se vs. HN5-CK comparison, the only pathway significantly enriched for both DEGs and DAMs was Phenylpropanoid biosynthesis (**Figures 8A, B**; **Supplementary Table S16**), a critical intermediary in the anthocyanin biosynthesis pathway, forming based on the chalcone structure. In contrast, HN2025-Se vs. HN2025-CK showed significant enrichment in multiple pathways, including alpha-Linolenic acid metabolism, Linoleic acid metabolism, Phenylalanine, tyrosine, and tryptophan biosynthesis, and the Biosynthesis of various plant secondary metabolites (**Figures 8C, D**; **Supplementary Table S17**).

Specifically, in HN2025-Se vs. HN2025-CK, there was notable upregulation of three PALs (Zm00001eb247620, Zm00001eb185240, Zm00001eb077230) alongside metabolites such as (E)-3-(2,3-Dihydroxyphenyl)-2-propenoic acid, L-Phenylalanine, 3-(3-Hydroxyphenyl)propanoic Acid, N-Acetyl-L-phenylalanine, and 4-Hydroxyphenylacetic Acid, all enriched in the phenylalanine metabolism pathway. Additionally, two PALs (Zm00001eb247620, Zm00001eb077230) and metabolites including 1-O-Sinapoyl-beta-D-glucose, L-Phenylalanine, Sinapic Acid, and 5-Hydroxyferulic Acid were enriched in phenylpropanoid biosynthesis. Consistent upregulation of C4H (Zm00001eb240910) and metabolites such as Naringenin Chalcone, Luteolin, Leucopelargonidin, Apigenin, Naringenin, Hesperetin, and Leucocyanidin were enriched in the flavonoid biosynthesis pathway (**Supplementary Table S18**). In conclusion, the upregulation of specific genes in HN2025 following selenium treatment appears to drive the increased metabolite content observed in anthocyanin biosynthesis. Notably, as upstream pathways of anthocyanin biosynthesis, the phenylalanine metabolism and flavonoid metabolism pathways show significant co-enrichment of DEGs and DAMs, suggesting a coordinated response to selenium treatment that enhances anthocyanin production in HN2025.

### qRT-PCR verification of DEGs

**Figure 9:**
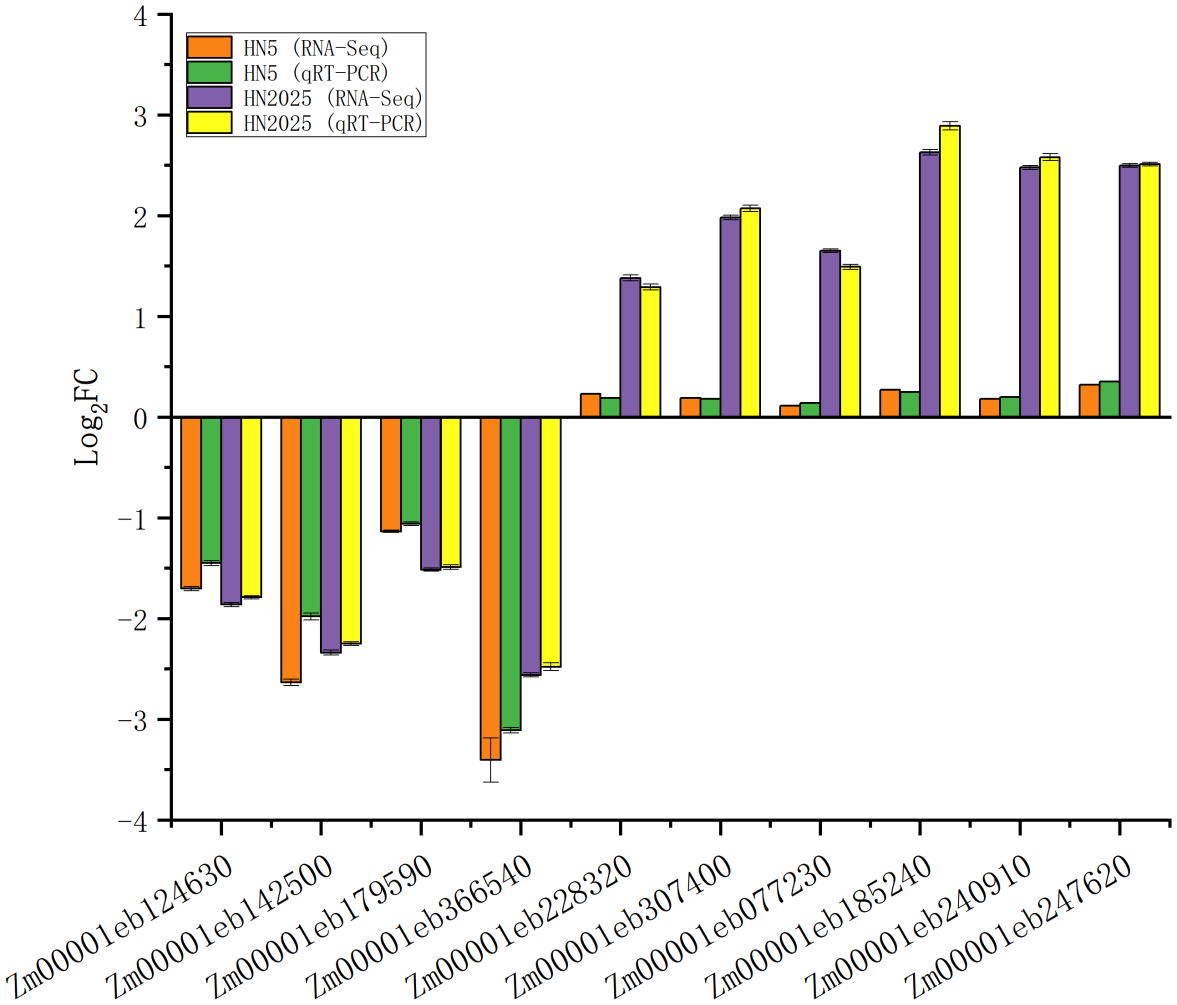
Quantitative real-time PCR (qRT-PCR) assay was carried out for lipid metabolism-related DEGs in HN5 and HN2025.

To evaluate the authenticity and reliability of DEGs identified in our transcriptomic data, we performed qRT-PCR validation on 10 selected DEGs (**Figure 9**), including three PAL (*Zm00001eb077230*, *Zm00001eb185240*, *Zm00001eb247620*) and one C4H (*Zm00001eb240910*), which are critical in the anthocyanin biosynthesis pathway, as well as four MYB (*Zm00001eb124630*, *Zm00001eb366540*, *Zm00001eb148320*, *Zm00001eb307400*) and two bHLH (*Zm00001eb142500*, *Zm00001eb179590*). qRT-PCR analysis showed that the expression of four transcription factors (*Zm00001eb124630*, *Zm00001eb142500*, *Zm00001eb179590*, *Zm00001eb366540*) in both HN5 and HN2025 was down-regulated after selenium treatment. This is consistent with the results of transcriptome analysis. Whereas qRT-PCR data for the other two transcription factors, three PALs and one C4H, were all significantly differentially expressed between HN5 and HN2025, which perfectly matches the transcriptome results.

## Discussion

Despite substantial advancements in elucidating the molecular mechanisms underpinning plant color formation (Coen and Meyerowitz, 1991; Wang et al., 2021), the regulation of pigments remains highly species-specific. Plant coloration is influenced by a complex interplay of environmental and genetic factors (Ausín et al., 2005). This study demonstrates that varying concentrations of selenium treatments significantly increased both total and organic selenium contents in the kernels of HN5 and HN2025. Notably, HN2025 exhibited a significantly higher selenium enrichment capacity compared to HN5. The anthocyanin content in HN5 was significantly elevated only under high selenium concentrations, whereas HN2025, which inherently possesses a high anthocyanin content, showed significant changes in anthocyanin levels primarily under the SE2 treatment at the S5 growth stage. This treatment also resulted in the highest percentage of organic selenium in both varieties. The findings suggest that the SE2 concentration at the S5 period effectively enhances both selenium and anthocyanin contents in maize kernels. These results indicate that HN2025, with its high anthocyanin content, has a superior capacity for selenium enrichment, corroborating previous findings (Pu et al., 2021). This study provides crucial insights into the differential responses of maize varieties to selenium treatment, highlighting the potential of targeted selenium applications in enhancing selenium and anthocyanin accumulation in maize kernels.

Anthocyanins, a class of flavonoids, are ubiquitous in various plant leaves, fruits, and other tissues and organs, renowned for their potent antioxidant properties (Tanaka et al., 2008; Petrussa et al., 2013). The glutathione metabolism pathway in plants is pivotal for antioxidant defense and anthocyanin synthesis under stress conditions by regulating reactive oxygen species (ROS) levels (Sharma et al., 2024). The glutathione S-transferase (GST) family facilitates the intracellular transport and accumulation of anthocyanins by forming complexes with these pigments (Manzoor et al., 2023). Amino acid metabolism contributes to anthocyanin synthesis by providing essential carbon skeletons and energy (Noctor and Foyer, 1998; Häusler et al., 2002). Phenylpropanoids serve as crucial precursors in the anthocyanin biosynthesis process (Dixon and Paiva, 1995), directly participating in the biosynthetic pathway (Vogt, 2010). Furthermore, flavonoids and related compounds play critical roles in anthocyanin synthesis; through a series of enzymatic reactions, these intermediates are converted into final anthocyanin products (Holton and Cornish, 1995; Winkel-Shirley, 2001; Tanaka et al., 2008). In this study, DEGs in both maize varieties were enriched in pathways associated with glutathione metabolism, amino acid metabolism, phenylpropanoid metabolism, and flavonoid metabolism. However, the number of DEGs enriched in these pathways was lower in HN5, which has a lower anthocyanin content, compared to HN2025. This suggests that selenium treatment induced the expression of a greater number of metabolic pathways related to anthocyanin synthesis in HN2025. Additionally, a greater number of differentially expressed transcription factors were detected in HN2025 compared to HN5, with 31 transcription factors specifically expressed in HN2025. Among these transcription factors were members of the MYB and bHLH families, which are known to play critical roles in regulating anthocyanin synthesis in maize (Riaz et al., 2019). Furthermore, several structural genes in the cyanidin biosynthesis pathway, such as PAL, CHS, and C4H, showed significant changes in expression levels following selenium treatment. These findings imply that anthocyanin synthesis is influenced by exogenous selenium intervention. Therefore, this research underscores the complex regulatory networks involved in anthocyanin biosynthesis and highlights the potential of selenium treatments to modulate these pathways for enhanced anthocyanin production.

Selenium treatment influences anthocyanin synthesis in plants by modulating the expression of key genes involved in the anthocyanin biosynthesis pathway. For example, in purple lettuce, selenite treatment up-regulates genes such as UDP-glucose flavonoid glycosyltransferase (UFGT) and flavanone 3-hydroxylase (F3H), which correlates with increased anthocyanin content (Liu et al., 2017). In rice, the anthocyanin biosynthesis pathway includes several crucial enzymes, such as phenylalanine ammonia-lyase (PAL), cinnamate 4-hydroxylase (C4H), anthocyanidin synthase (ANS), and UFGT, which are directly involved in anthocyanin synthesis (Mackon et al., 2021). The influence of selenium on these pathways can vary depending on the plant species and the selenium concentration used. While selenium often promotes anthocyanin accumulation by up-regulating related genes, it can also lead to the down-regulation of certain genes, depending on experimental conditions and plant species (Mackon et al., 2021). In this study, HN2025, a high-anthocyanin content maize variety, showed greater sensitivity to selenium treatment. Gene Ontology (GO) annotation revealed 2,415 DEGs in HN2025, three times more than in HN5. Unique GO terms for HN2025 included L-phenylalanine catabolic process, cinnamic acid metabolic process, and regulation of phenylpropanoid metabolic process. However, KEGG enrichment analysis identified anthocyanin synthesis-related metabolic pathways in both varieties, such as flavonoid biosynthesis, phenylalanine biosynthesis, flavone and flavonol biosynthesis, and phenylpropanoid biosynthesis. Notably, structural genes related to anthocyanin synthesis were found exclusively in HN2025, indicating a more profound influence of selenium treatment on anthocyanin synthesis in high-content varieties.

The metabolomic analysis further demonstrated that HN2025 produced 140 more metabolites than HN5 after selenium treatment, including key substances in the anthocyanin biosynthesis pathway such as naringin, anthocyanins, kaempferol, cinnamic acid, L-phenylalanine, and chalcone intermediates. Despite these differences, the highest enrichment factors were not observed for these substances. Significant differences in selenium-induced stress responses were noted between the two varieties, with DEGs and DAMs annotated in anthocyanin synthesis-related pathways. In HN5, significant enrichment was observed only in the phenylpropanoid biosynthesis pathway. In contrast, HN2025 showed significant enrichment in α-linolenic acid metabolism, linoleic acid metabolism, phenylalanine, tyrosine, and tryptophan biosynthesis, and biosynthesis of various plant secondary metabolites. This includes the consistent enrichment of three PAL genes and metabolites such as (E)-3-(2,3-dihydroxy phenyl)-2-propenoic acid, L-phenylalanine, 3-(3-hydroxyphenyl)propanoic acid, N-acetyl-L-phenylalanine, and 4-hydroxyphenylacetic acid in phenylalanine metabolism. Additionally, two PAL genes and metabolites 1-O-sinapoyl-beta-D-glucose and L-phenylalanine were enriched in the phenylpropanoid biosynthesis pathway. Furthermore, C4H (*Zm00001eb240910*) and metabolites such as naringenin chalcone, naringenin, hesperetin, and tricin were significantly enriched in the flavonoid biosynthesis pathway. We observed changes in anthocyanin content and physiological indices in HN5 and HN2025 grains before and after selenium treatment. Considering these transcriptional and metabolic differences, we propose that the color variation in maize grains may be associated with the levels of these derivatives induced by selenium treatment. These findings highlight the intricate regulatory networks governing anthocyanin biosynthesis and the potential of selenium treatments to enhance anthocyanin production in maize.

## Conclusions

By integrating transcriptomic and metabolomic analyses, we elucidated that anthocyanin content in maize kernels is positively correlated with their selenium enrichment capacity. Maize varieties exhibiting high selenium enrichment capacity demonstrated pronounced sensitivity to selenium stress at both transcriptional and metabolic levels. Specifically, genes and transcription factors associated with anthocyanin biosynthesis, flavonoid and flavonol biosynthesis, glutathione metabolism, phenylalanine biosynthesis, and phenylalanine metabolism were markedly induced in maize varieties with elevated anthocyanin content. It was further found that three upregulated phenylalanine ammonia lyase (PAL) genes and one cinnamate 4-hydroxylase (C4H) gene, which are inextricably linked to anthocyanin synthesis, as well as DAMs were significantly enriched. These findings highlight the complex regulatory network of maize anthocyanin biosynthesis under selenium stress. This study lays the foundation for future studies of anthocyanin biosynthesis and regulatory mechanisms in maize under selenium stress and provides potential targets for genetic and agronomic interventions to enhance anthocyanin production.

## Data Availability

All raw sequencing data have been deposited at the NCBI Sequence Read Archive Archive (https://www.ncbi.nlm.nih.gov/sra; PRJNA1129326).
